# 2-[3-(1*H*-Benzimidazol-2-yl)prop­yl]-1*H*-benzimidazol-3-ium 3,5-dicarb­oxy­benzoate–benzene-1,3,5-tricarb­oxy­lic acid–water (1/1/1)

**DOI:** 10.1107/S1600536810039139

**Published:** 2010-10-09

**Authors:** Guo-dong Feng, Luan Jiang

**Affiliations:** a Department of Chemistry and Chemical Engineering, Baoji University of Arts and Sciences, Baoji, Shaanxi 721007, People’s Republic of China

## Abstract

The title compound, C_17_H_17_N_4_^+^·C_9_H_5_O_6_^−^·C_9_H_6_O_6_·H_2_O, contains a protonated 2,2′-(1,3-propanedi­yl)bis­(1*H*-benzimidazole) cation, a deprotonated benzene-1,3,5-tricarb­oxy­lic acid anion, a neutral benzene-1,3,5-tricarb­oxy­lic acid mol­ecule and a water mol­ecule, which are linked together through N—H⋯O, O—H⋯O and weak C—H⋯O hydrogen bonds into almost double sheets parallel to (4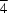 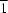). These hydrogen-bonded sheets are packed in the crystal with the formation of centrosymmetric voids of 25.5 Å^3^, which are filled by the water mol­ecules.

## Related literature

For the coordination chemistry of bis-benzimidazoles, see: Sun *et al.* (2010 ▶). For the clinical applications of the benzimidazole ring system, see Harrell *et al.* (2004 ▶). For novel proton-transfer compounds, see Aghabozorg *et al.* (2008 ▶). For applications of benzimidazole and bis-benzimidazole compounds, see: Chang *et al.* (2008 ▶). 
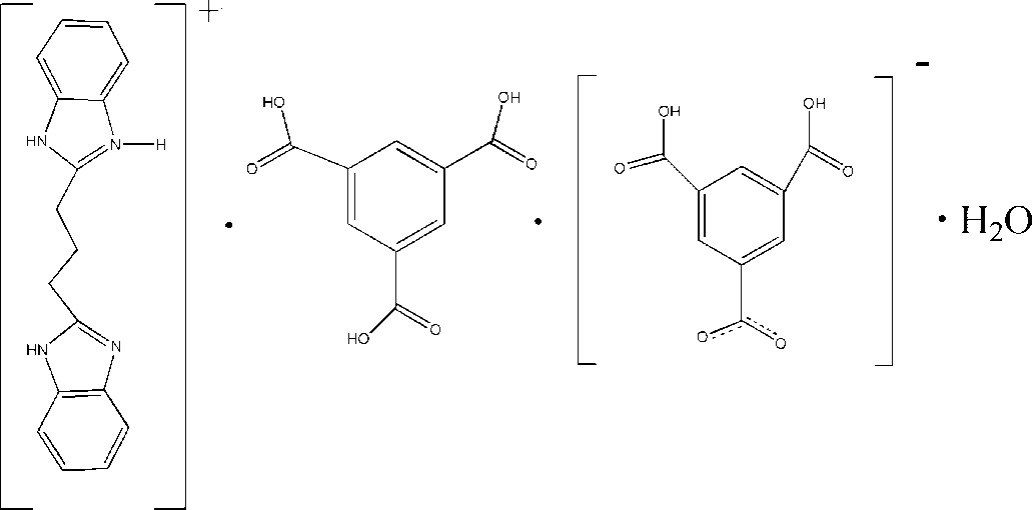


## Experimental

### 

#### Crystal data

C_17_H_17_N_4_^+^·C_9_H_5_O_6_^−^·C_9_H_6_O_6_·H_2_O
*M* *_r_* = 714.63
Triclinic, 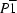

*a* = 8.7711 (3) Å
*b* = 10.8389 (6) Å
*c* = 17.2999 (9) Å
α = 81.520 (5)°
β = 84.131 (4)°
γ = 86.998 (4)°
*V* = 1617.02 (13) Å^3^
*Z* = 2
Mo *K*α radiation
μ = 0.11 mm^−1^
*T* = 293 K
0.4 × 0.32 × 0.2 mm

#### Data collection

Bruker APEX CCD area-detector diffractometer
Absorption correction: multi-scan (*SADABS*; Sheldrick, 1996 ▶) *T*_min_ = 0.957, *T*_max_ = 0.977
9922 measured reflections
5657 independent reflections
3630 reflections with *I* > 2σ(*I*)
*R*_int_ = 0.023

#### Refinement

*R*[*F* ^2^ > 2σ(*F* ^2^)] = 0.039
*wR*(*F* ^2^) = 0.081
*S* = 1.07
5657 reflections
469 parameters
3 restraints
H-atom parameters constrained
Δρ_max_ = 0.28 e Å^−3^
Δρ_min_ = −0.22 e Å^−3^

### 

Data collection: *SMART* (Bruker, 2005 ▶); cell refinement: *SAINT* (Bruker, 2005 ▶); data reduction: *SAINT*; program(s) used to solve structure: *SHELXS97* (Sheldrick, 2008 ▶); program(s) used to refine structure: *SHELXL97* (Sheldrick, 2008 ▶); molecular graphics: *SHELXTL-Plus* (Sheldrick, 2008 ▶); software used to prepare material for publication: *SHELXL97*.

## Supplementary Material

Crystal structure: contains datablocks I, global. DOI: 10.1107/S1600536810039139/pb2041sup1.cif

Structure factors: contains datablocks I. DOI: 10.1107/S1600536810039139/pb2041Isup2.hkl

Additional supplementary materials:  crystallographic information; 3D view; checkCIF report

## Figures and Tables

**Table 1:** Hydrogen-bond geometry (Å, °)

*D*—H⋯*A*	*D*—H	H⋯*A*	*D*⋯*A*	*D*—H⋯*A*
O1—H1⋯O10^i^	0.82	1.82	2.6404 (16)	178
O9—H9⋯O2^i^	0.82	1.85	2.6682 (16)	177
N1—H1*A*⋯O11	0.86	1.84	2.6560 (17)	159
N4—H4*A*⋯O3	0.86	2.05	2.8435 (17)	153
C1—H221⋯O10	0.93	2.51	3.414 (2)	163
C9—H9*A*⋯O5^ii^	0.97	2.44	3.346 (2)	156

Symmetry codes: (i) 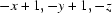; (ii) 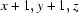.

**Fig. 1.**
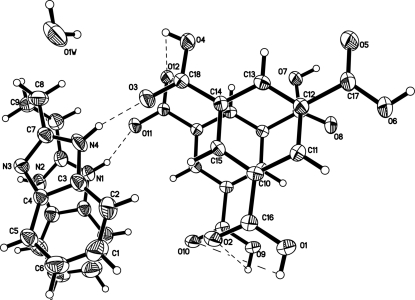
The structure of the title compound in 50% probability ellipsoids. The O—H···O hydrogen bonds link the two benzene-1,3,5-tricarboxylic acids- units and N—H···O and weak C—H···O hydrogen bonds link bis-benzimidazoles cations to these chains.

**Fig. 2.**
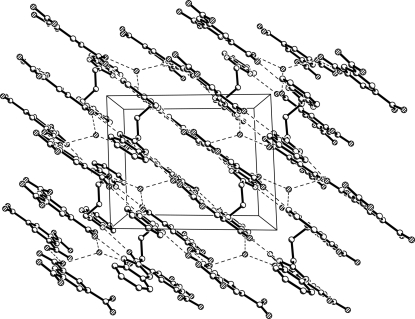
A view along the c axis of the crystal packing of the title compound.

## supplementary crystallographic information

### Comment

Bis-benzimidazoles are known to be strong chelating agents coordinating through both of the C=N group nitrogen atoms (Sun *et al.*, 2010). Recently, The benzimidazole ring system is present in clinically approved anthelmintics, antiulcers, antivirals, and antihistamines (Harrell, *et al.*, 2004).

A number of cases were reported in which a proton transferred from a carboxylic acid to an amine to form some novel proton transfer compounds (Aghabozorg *et al.*, 2008). In this work, we report a new proton transfer compound obtained from benzene-1,3,5-tricarboxylic acid as a proton donor and bis-benzimidazoles as an acceptor.

The crystal structure of the title proton transfer compound shows that a single proton from one of the carboxyl groups of the benzene-1,3,5-tricarboxylic acids was transferred to the N-ring atom of benzimidazoles. On the other hand, an interesting feature exhibited by the crystal structure is that just one benzene-1,3,5-tricarboxylic acids as a proton donor and another benzene-1,3,5-tricarboxylic acids is in an un-ionized state. The two benzene-1,3,5-tricarboxylic acids are parallel. In the crystal structure, intermolecular N—H···O,*O*—H···O and weak C—H···O hydrogen bonds (Table 1) link cations and anions into double-planar parallel to the (4,-4,-1) plane. These hydrogen-bonded sheets are further packed into crystal with the formation of centrosymmetric voids of 25.5 Å3, which are filled by the disordered water molecules.

### Experimental

The compound was prepared by a hydrothermal method. A mixture of 2,2'-(1,3-propanediyl)bis(1*H*-benzimidazole)(0.5 mmol), benzene-1,3,5-tricarboxylic acid (0.6 mmol),and water (10 ml) was stirred for 20 min and then transferred to a 23 ml Teflon reactor. The reactor was kept at 433 K for 72 h under autogenous pressure. Single crystals were obtained after cooling to room temperature.

### Refinement

All H atoms were placed in calculated positions and refined in a riding-model approximation with; C—H = 0.95–0.99 Å, N—H = 0.88 Å, O—H = 0.83–0.85Å and *U*_iso_(H) = 1.2 *U*_eq_(C) or *U*_iso_(H) = 1.5 *U*_eq_(O).

### Crystal data

**Table:**

C_17_H_17_N_4_^+^·C_9_H_5_O_6_^−^·C_9_H_6_O_6_·H_2_O	*Z* = 2
*M**_r_* = 714.63	*F*(000) = 744
Triclinic, *P*1	*D*_x_ = 1.468 Mg m^−^^3^
*a* = 8.7711 (3) Å	Mo *K*α radiation, λ = 0.71073 Å
*b* = 10.8389 (6) Å	Cell parameters from 165 reflections
*c* = 17.2999 (9) Å	θ = 2.8–23.6°
α = 81.520 (5)°	µ = 0.11 mm^−^^1^
β = 84.131 (4)°	*T* = 293 K
γ = 86.998 (4)°	Block, colorless
*V* = 1617.02 (13) Å^3^	0.4 × 0.32 × 0.2 mm

### Data collection

**Table:**

Bruker APEX CCD area-detector diffractometer	5657 independent reflections
Radiation source: fine-focus sealed tube	3630 reflections with *I* > 2σ(*I*)
graphite	*R*_int_ = 0.023
φ and ω scans	θ_max_ = 25.0°, θ_min_ = 3.3°
Absorption correction: multi-scan (*SADABS*; Sheldrick, 1996)	*h* = −10→9
*T*_min_ = 0.957, *T*_max_ = 0.977	*k* = −12→12
9922 measured reflections	*l* = −20→20

### Refinement

**Table:**

Refinement on *F*^2^	Primary atom site location: structure-invariant direct methods
Least-squares matrix: full	Secondary atom site location: difference Fourier map
*R*[*F*^2^ > 2σ(*F*^2^)] = 0.039	Hydrogen site location: inferred from neighbouring sites
*wR*(*F*^2^) = 0.081	H-atom parameters constrained
*S* = 1.07	*w* = 1/[σ^2^(*F*_o_^2^) + (0.0349*P*)^2^] where *P* = (*F*_o_^2^ + 2*F*_c_^2^)/3
5657 reflections	(Δ/σ)_max_ < 0.001
469 parameters	Δρ_max_ = 0.28 e Å^−^^3^
3 restraints	Δρ_min_ = −0.22 e Å^−^^3^

### Special details

**Table:**

Geometry. All e.s.d.'s (except the e.s.d. in the dihedral angle between two l.s. planes) are estimated using the full covariance matrix. The cell e.s.d.'s are taken into account individually in the estimation of e.s.d.'s in distances, angles and torsion angles; correlations between e.s.d.'s in cell parameters are only used when they are defined by crystal symmetry. An approximate (isotropic) treatment of cell e.s.d.'s is used for estimating e.s.d.'s involving l.s. planes.
Refinement. Refinement of *F*^2^ against ALL reflections. The weighted *R*-factor *wR* and goodness of fit *S* are based on *F*^2^, conventional *R*-factors *R* are based on *F*, with *F* set to zero for negative *F*^2^. The threshold expression of *F*^2^ > σ(*F*^2^) is used only for calculating *R*-factors(gt) *etc*. and is not relevant to the choice of reflections for refinement. *R*-factors based on *F*^2^ are statistically about twice as large as those based on *F*, and *R*- factors based on ALL data will be even larger.

### Fractional atomic coordinates and isotropic or equivalent isotropic displacement parameters (Å^2^)

**Table:**

	*x*	*y*	*z*	*U*_iso_*/*U*_eq_	
C1	0.9366 (2)	0.70532 (19)	0.12984 (12)	0.0472 (5)	
H221	0.8725	0.6395	0.1302	0.057*	
C2	0.9969 (2)	0.7728 (2)	0.06116 (13)	0.0597 (6)	
H222	0.9733	0.7522	0.0136	0.072*	
C3	1.0926 (2)	0.8712 (2)	0.06101 (13)	0.0651 (7)	
H223	1.1301	0.9150	0.0131	0.078*	
C4	1.1335 (2)	0.9060 (2)	0.12862 (13)	0.0542 (6)	
H224	1.1979	0.9716	0.1281	0.065*	
C5	1.07310 (18)	0.83745 (17)	0.19804 (11)	0.0372 (5)	
C6	0.97641 (18)	0.74056 (16)	0.19828 (11)	0.0341 (4)	
C7	0.99913 (18)	0.76278 (17)	0.32189 (11)	0.0355 (4)	
C8	0.9711 (2)	0.74754 (18)	0.40866 (11)	0.0433 (5)	
H8A	1.0687	0.7459	0.4307	0.052*	
H8B	0.9248	0.6679	0.4271	0.052*	
C9	0.8672 (2)	0.85092 (18)	0.43867 (11)	0.0428 (5)	
H9A	0.9056	0.9307	0.4135	0.051*	
H9B	0.8744	0.8465	0.4946	0.051*	
C10	0.6983 (2)	0.8473 (2)	0.42501 (10)	0.0458 (5)	
H10A	0.6598	0.7672	0.4496	0.055*	
H10B	0.6412	0.9114	0.4508	0.055*	
C11	0.66781 (17)	0.86607 (16)	0.34136 (10)	0.0325 (4)	
C12	0.57954 (18)	0.83069 (16)	0.23153 (10)	0.0332 (4)	
C13	0.5143 (2)	0.7843 (2)	0.17270 (12)	0.0523 (6)	
H13	0.4556	0.7137	0.1832	0.063*	
C14	0.5407 (2)	0.8473 (2)	0.09827 (13)	0.0579 (6)	
H14	0.4997	0.8184	0.0571	0.069*	
C15	0.6273 (2)	0.9538 (2)	0.08258 (11)	0.0501 (5)	
H15	0.6404	0.9955	0.0315	0.060*	
C16	0.69382 (19)	0.99856 (17)	0.14092 (10)	0.0376 (5)	
H16	0.7530	1.0689	0.1302	0.045*	
C17	0.66929 (17)	0.93482 (15)	0.21629 (10)	0.0278 (4)	
C18	0.46437 (16)	0.20168 (15)	0.29690 (9)	0.0248 (4)	
C19	0.49709 (17)	0.24146 (15)	0.21723 (9)	0.0283 (4)	
H19	0.4573	0.2003	0.1807	0.034*	
C20	0.58961 (18)	0.34311 (16)	0.19206 (9)	0.0295 (4)	
C21	0.64414 (18)	0.40664 (16)	0.24701 (9)	0.0303 (4)	
H21	0.7039	0.4756	0.2300	0.036*	
C22	0.61089 (17)	0.36895 (15)	0.32672 (9)	0.0260 (4)	
C23	0.52217 (17)	0.26558 (15)	0.35086 (9)	0.0273 (4)	
H23	0.5009	0.2384	0.4042	0.033*	
C24	0.37076 (18)	0.08932 (16)	0.32472 (10)	0.0288 (4)	
C25	0.6362 (2)	0.38696 (18)	0.10842 (10)	0.0380 (5)	
C26	0.67101 (18)	0.43956 (16)	0.38498 (10)	0.0298 (4)	
C27	0.18279 (17)	0.44108 (16)	0.18951 (9)	0.0294 (4)	
C28	0.08562 (18)	0.34199 (16)	0.21251 (10)	0.0315 (4)	
H28	0.0461	0.3032	0.1748	0.038*	
C29	0.04780 (17)	0.30111 (15)	0.29153 (9)	0.0278 (4)	
C30	0.10540 (17)	0.36020 (15)	0.34742 (9)	0.0282 (4)	
H30	0.0790	0.3333	0.4004	0.034*	
C31	0.20262 (17)	0.45961 (15)	0.32504 (9)	0.0248 (4)	
C32	0.23957 (17)	0.49858 (15)	0.24616 (10)	0.0294 (4)	
H32	0.3041	0.5650	0.2307	0.035*	
C33	0.2313 (2)	0.48724 (17)	0.10617 (10)	0.0380 (5)	
C34	−0.05308 (18)	0.19177 (16)	0.31858 (10)	0.0325 (4)	
C35	0.27119 (17)	0.52423 (16)	0.38307 (10)	0.0287 (4)	
N1	0.93470 (15)	0.69651 (13)	0.27654 (8)	0.0348 (4)	
H1A	0.8758	0.6353	0.2931	0.042*	
N2	1.08535 (15)	0.84712 (14)	0.27610 (9)	0.0402 (4)	
H2A	1.1402	0.8996	0.2925	0.048*	
N3	0.72286 (14)	0.95552 (13)	0.28622 (8)	0.0305 (3)	
N4	0.57988 (15)	0.79119 (14)	0.31099 (9)	0.0375 (4)	
H4A	0.5316	0.7288	0.3369	0.045*	
O1	0.16654 (16)	0.43594 (14)	0.05506 (7)	0.0646 (5)	
H1	0.1992	0.4662	0.0106	0.097*	
O2	0.32635 (14)	0.56827 (13)	0.08772 (7)	0.0491 (4)	
O3	0.34628 (14)	0.61690 (12)	0.36169 (7)	0.0446 (3)	
O4	0.24293 (14)	0.47490 (11)	0.45530 (7)	0.0460 (4)	
H4	0.2837	0.5149	0.4837	0.069*	
O5	−0.08461 (16)	0.15746 (13)	0.38823 (7)	0.0574 (4)	
O6	−0.09809 (13)	0.14016 (12)	0.26303 (7)	0.0464 (4)	
H6	−0.1521	0.0816	0.2819	0.070*	
O7	0.37661 (14)	0.05198 (12)	0.40002 (7)	0.0461 (4)	
H7	0.3245	−0.0096	0.4136	0.069*	
O8	0.30023 (13)	0.03803 (12)	0.28260 (7)	0.0425 (3)	
O9	0.57854 (15)	0.32907 (13)	0.05788 (7)	0.0555 (4)	
H9	0.6104	0.3591	0.0132	0.083*	
O10	0.72506 (17)	0.47175 (14)	0.08869 (7)	0.0657 (5)	
O11	0.74251 (14)	0.53540 (12)	0.36033 (7)	0.0474 (4)	
O12	0.64331 (14)	0.39760 (11)	0.45685 (7)	0.0428 (3)	
O1W	0.27197 (15)	0.84782 (13)	0.47477 (8)	0.0601 (4)	
H2	0.2094	0.8558	0.5152	0.090*	
H3	0.3126	0.7750	0.4838	0.090*	

### Atomic displacement parameters (Å^2^)

**Table:**

	*U*^11^	*U*^22^	*U*^33^	*U*^12^	*U*^13^	*U*^23^
C1	0.0492 (11)	0.0476 (13)	0.0471 (13)	−0.0132 (10)	0.0003 (10)	−0.0137 (11)
C2	0.0618 (13)	0.0745 (17)	0.0432 (13)	−0.0145 (12)	0.0032 (11)	−0.0119 (12)
C3	0.0638 (14)	0.0800 (19)	0.0451 (14)	−0.0162 (13)	0.0106 (12)	0.0061 (13)
C4	0.0456 (12)	0.0538 (15)	0.0588 (15)	−0.0215 (10)	0.0096 (11)	0.0035 (12)
C5	0.0306 (9)	0.0390 (12)	0.0417 (12)	−0.0072 (8)	−0.0007 (8)	−0.0050 (9)
C6	0.0307 (9)	0.0310 (11)	0.0399 (11)	−0.0049 (8)	0.0011 (8)	−0.0046 (9)
C7	0.0315 (9)	0.0315 (11)	0.0436 (12)	−0.0020 (8)	−0.0067 (9)	−0.0036 (9)
C8	0.0480 (11)	0.0414 (12)	0.0416 (12)	−0.0058 (9)	−0.0133 (9)	−0.0020 (10)
C9	0.0619 (12)	0.0398 (12)	0.0281 (10)	−0.0050 (9)	−0.0125 (9)	−0.0035 (9)
C10	0.0516 (12)	0.0543 (14)	0.0297 (11)	−0.0072 (10)	0.0017 (9)	−0.0022 (10)
C11	0.0299 (9)	0.0319 (11)	0.0340 (10)	−0.0047 (8)	0.0019 (8)	−0.0018 (9)
C12	0.0310 (9)	0.0316 (11)	0.0363 (11)	−0.0096 (8)	−0.0026 (8)	−0.0001 (9)
C13	0.0524 (12)	0.0532 (14)	0.0553 (14)	−0.0302 (10)	−0.0108 (11)	−0.0065 (12)
C14	0.0576 (13)	0.0755 (17)	0.0466 (14)	−0.0257 (12)	−0.0172 (11)	−0.0122 (12)
C15	0.0509 (11)	0.0655 (15)	0.0343 (11)	−0.0164 (11)	−0.0110 (10)	0.0019 (11)
C16	0.0383 (10)	0.0351 (11)	0.0385 (11)	−0.0128 (8)	−0.0038 (9)	0.0022 (9)
C17	0.0280 (9)	0.0264 (10)	0.0296 (10)	−0.0052 (7)	−0.0048 (8)	−0.0030 (8)
C18	0.0283 (9)	0.0249 (10)	0.0211 (9)	−0.0041 (7)	−0.0012 (7)	−0.0029 (7)
C19	0.0336 (9)	0.0290 (10)	0.0243 (10)	−0.0061 (7)	−0.0041 (7)	−0.0080 (8)
C20	0.0372 (9)	0.0306 (10)	0.0208 (9)	−0.0097 (8)	−0.0005 (8)	−0.0026 (8)
C21	0.0360 (9)	0.0295 (10)	0.0251 (10)	−0.0127 (8)	−0.0005 (8)	−0.0007 (8)
C22	0.0310 (9)	0.0263 (10)	0.0213 (9)	−0.0068 (7)	−0.0019 (7)	−0.0033 (8)
C23	0.0334 (9)	0.0280 (10)	0.0203 (9)	−0.0058 (7)	−0.0021 (7)	−0.0017 (8)
C24	0.0326 (9)	0.0288 (10)	0.0251 (10)	−0.0064 (8)	0.0020 (8)	−0.0058 (8)
C25	0.0524 (11)	0.0419 (12)	0.0214 (10)	−0.0198 (9)	−0.0023 (9)	−0.0046 (9)
C26	0.0371 (9)	0.0282 (11)	0.0249 (10)	−0.0097 (8)	−0.0030 (8)	−0.0036 (8)
C27	0.0352 (9)	0.0312 (11)	0.0215 (9)	−0.0090 (8)	−0.0015 (8)	−0.0009 (8)
C28	0.0362 (9)	0.0338 (11)	0.0262 (10)	−0.0100 (8)	−0.0049 (8)	−0.0061 (8)
C29	0.0314 (9)	0.0278 (10)	0.0240 (9)	−0.0078 (7)	−0.0036 (7)	−0.0005 (8)
C30	0.0335 (9)	0.0283 (10)	0.0219 (9)	−0.0067 (7)	−0.0011 (7)	0.0003 (8)
C31	0.0289 (8)	0.0228 (10)	0.0229 (9)	−0.0049 (7)	−0.0030 (7)	−0.0026 (7)
C32	0.0348 (9)	0.0261 (10)	0.0267 (10)	−0.0121 (8)	−0.0005 (8)	0.0008 (8)
C33	0.0493 (11)	0.0399 (12)	0.0260 (10)	−0.0186 (9)	0.0004 (9)	−0.0050 (9)
C34	0.0348 (10)	0.0315 (11)	0.0327 (12)	−0.0121 (8)	−0.0051 (8)	−0.0041 (9)
C35	0.0325 (9)	0.0283 (10)	0.0252 (10)	−0.0069 (8)	−0.0006 (8)	−0.0029 (8)
N1	0.0359 (8)	0.0276 (9)	0.0409 (10)	−0.0117 (7)	−0.0010 (7)	−0.0031 (7)
N2	0.0348 (8)	0.0367 (10)	0.0515 (11)	−0.0159 (7)	−0.0061 (7)	−0.0071 (8)
N3	0.0339 (8)	0.0300 (9)	0.0275 (8)	−0.0087 (6)	−0.0005 (6)	−0.0022 (7)
N4	0.0376 (8)	0.0312 (9)	0.0417 (10)	−0.0166 (7)	0.0004 (7)	0.0035 (7)
O1	0.0948 (11)	0.0814 (11)	0.0217 (7)	−0.0564 (9)	−0.0007 (7)	−0.0055 (7)
O2	0.0662 (8)	0.0569 (9)	0.0247 (7)	−0.0361 (7)	0.0015 (6)	0.0000 (6)
O3	0.0600 (8)	0.0431 (8)	0.0326 (7)	−0.0306 (7)	−0.0048 (6)	−0.0016 (6)
O4	0.0741 (9)	0.0448 (9)	0.0225 (7)	−0.0326 (7)	−0.0075 (6)	−0.0034 (6)
O5	0.0845 (10)	0.0606 (10)	0.0279 (8)	−0.0454 (8)	0.0000 (7)	0.0023 (7)
O6	0.0582 (8)	0.0465 (9)	0.0372 (8)	−0.0322 (7)	−0.0033 (6)	−0.0048 (7)
O7	0.0704 (9)	0.0426 (8)	0.0260 (7)	−0.0324 (7)	−0.0022 (6)	0.0016 (6)
O8	0.0525 (7)	0.0419 (8)	0.0363 (7)	−0.0251 (6)	−0.0087 (6)	−0.0050 (6)
O9	0.0903 (10)	0.0597 (10)	0.0190 (7)	−0.0410 (8)	−0.0007 (7)	−0.0041 (7)
O10	0.0969 (11)	0.0798 (11)	0.0235 (7)	−0.0626 (9)	0.0040 (7)	−0.0039 (7)
O11	0.0700 (9)	0.0423 (8)	0.0329 (7)	−0.0348 (7)	−0.0027 (6)	−0.0049 (6)
O12	0.0710 (8)	0.0401 (8)	0.0200 (7)	−0.0265 (7)	−0.0052 (6)	−0.0040 (6)
O1W	0.0641 (8)	0.0412 (9)	0.0669 (10)	−0.0139 (7)	0.0056 (7)	0.0145 (7)

### Geometric parameters (Å, °)

**Table:**

C1—C2	1.372 (3)	C19—H19	0.9300
C1—C6	1.379 (2)	C20—C21	1.388 (2)
C1—H221	0.9300	C20—C25	1.478 (2)
C2—C3	1.391 (3)	C21—C22	1.385 (2)
C2—H222	0.9300	C21—H21	0.9300
C3—C4	1.367 (3)	C22—C23	1.386 (2)
C3—H223	0.9300	C22—C26	1.503 (2)
C4—C5	1.386 (3)	C23—H23	0.9300
C4—H224	0.9300	C24—O8	1.2127 (19)
C5—N2	1.385 (2)	C24—O7	1.311 (2)
C5—C6	1.383 (2)	C25—O10	1.2232 (19)
C6—N1	1.386 (2)	C25—O9	1.304 (2)
C7—N1	1.323 (2)	C26—O11	1.2399 (19)
C7—N2	1.331 (2)	C26—O12	1.2628 (19)
C7—C8	1.482 (3)	C27—C32	1.382 (2)
C8—C9	1.524 (2)	C27—C28	1.392 (2)
C8—H8A	0.9700	C27—C33	1.481 (2)
C8—H8B	0.9700	C28—C29	1.385 (2)
C9—C10	1.528 (2)	C28—H28	0.9300
C9—H9A	0.9700	C29—C30	1.384 (2)
C9—H9B	0.9700	C29—C34	1.506 (2)
C10—C11	1.481 (2)	C30—C31	1.394 (2)
C10—H10A	0.9700	C30—H30	0.9300
C10—H10B	0.9700	C31—C32	1.378 (2)
C11—N3	1.329 (2)	C31—C35	1.495 (2)
C11—N4	1.343 (2)	C32—H32	0.9300
C12—N4	1.378 (2)	C33—O2	1.2277 (19)
C12—C13	1.384 (2)	C33—O1	1.302 (2)
C12—C17	1.388 (2)	C34—O5	1.215 (2)
C13—C14	1.369 (3)	C34—O6	1.2853 (19)
C13—H13	0.9300	C35—O3	1.2204 (18)
C14—C15	1.393 (3)	C35—O4	1.2893 (19)
C14—H14	0.9300	N1—H1A	0.8600
C15—C16	1.374 (2)	N2—H2A	0.8600
C15—H15	0.9300	N4—H4A	0.8600
C16—C17	1.384 (2)	O1—H1	0.8200
C16—H16	0.9300	O4—H4	0.8200
C17—N3	1.394 (2)	O6—H6	0.8200
C18—C19	1.387 (2)	O7—H7	0.8200
C18—C23	1.390 (2)	O9—H9	0.8200
C18—C24	1.497 (2)	O1W—H2	0.8561
C19—C20	1.394 (2)	O1W—H3	0.8490
			
C2—C1—C6	116.25 (18)	C20—C19—H19	120.0
C2—C1—H221	121.9	C21—C20—C19	119.67 (15)
C6—C1—H221	121.9	C21—C20—C25	117.19 (14)
C1—C2—C3	121.6 (2)	C19—C20—C25	123.13 (14)
C1—C2—H222	119.2	C22—C21—C20	121.04 (14)
C3—C2—H222	119.2	C22—C21—H21	119.5
C4—C3—C2	122.5 (2)	C20—C21—H21	119.5
C4—C3—H223	118.8	C23—C22—C21	118.60 (15)
C2—C3—H223	118.8	C23—C22—C26	121.43 (15)
C3—C4—C5	115.98 (18)	C21—C22—C26	119.98 (14)
C3—C4—H224	122.0	C22—C23—C18	121.36 (15)
C5—C4—H224	122.0	C22—C23—H23	119.3
C4—C5—N2	132.23 (17)	C18—C23—H23	119.3
C4—C5—C6	121.61 (18)	O8—C24—O7	124.20 (15)
N2—C5—C6	106.13 (15)	O8—C24—C18	124.19 (16)
C1—C6—N1	131.79 (16)	O7—C24—C18	111.60 (14)
C1—C6—C5	122.11 (17)	O10—C25—O9	122.68 (15)
N1—C6—C5	106.09 (15)	O10—C25—C20	121.29 (15)
N1—C7—N2	108.36 (16)	O9—C25—C20	116.02 (14)
N1—C7—C8	124.59 (16)	O11—C26—O12	123.93 (15)
N2—C7—C8	126.98 (17)	O11—C26—C22	118.80 (15)
C7—C8—C9	113.30 (16)	O12—C26—C22	117.27 (14)
C7—C8—H8A	108.9	C32—C27—C28	119.36 (15)
C9—C8—H8A	108.9	C32—C27—C33	117.75 (14)
C7—C8—H8B	108.9	C28—C27—C33	122.88 (15)
C9—C8—H8B	108.9	C29—C28—C27	120.05 (15)
H8A—C8—H8B	107.7	C29—C28—H28	120.0
C8—C9—C10	115.43 (15)	C27—C28—H28	120.0
C8—C9—H9A	108.4	C30—C29—C28	119.73 (14)
C10—C9—H9A	108.4	C30—C29—C34	118.75 (15)
C8—C9—H9B	108.4	C28—C29—C34	121.51 (15)
C10—C9—H9B	108.4	C29—C30—C31	120.69 (15)
H9A—C9—H9B	107.5	C29—C30—H30	119.7
C11—C10—C9	114.59 (14)	C31—C30—H30	119.7
C11—C10—H10A	108.6	C32—C31—C30	118.75 (14)
C9—C10—H10A	108.6	C32—C31—C35	118.50 (13)
C11—C10—H10B	108.6	C30—C31—C35	122.74 (14)
C9—C10—H10B	108.6	C27—C32—C31	121.41 (14)
H10A—C10—H10B	107.6	C27—C32—H32	119.3
N3—C11—N4	110.65 (15)	C31—C32—H32	119.3
N3—C11—C10	126.11 (16)	O2—C33—O1	123.21 (16)
N4—C11—C10	123.23 (15)	O2—C33—C27	121.45 (15)
N4—C12—C13	132.57 (16)	O1—C33—C27	115.34 (14)
N4—C12—C17	105.30 (15)	O5—C34—O6	124.88 (15)
C13—C12—C17	122.10 (17)	O5—C34—C29	120.36 (15)
C14—C13—C12	116.70 (17)	O6—C34—C29	114.75 (15)
C14—C13—H13	121.6	O3—C35—O4	124.28 (15)
C12—C13—H13	121.6	O3—C35—C31	120.99 (15)
C13—C14—C15	121.71 (19)	O4—C35—C31	114.73 (14)
C13—C14—H14	119.1	C7—N1—C6	109.82 (14)
C15—C14—H14	119.1	C7—N1—H1A	125.1
C16—C15—C14	121.43 (19)	C6—N1—H1A	125.1
C16—C15—H15	119.3	C7—N2—C5	109.58 (15)
C14—C15—H15	119.3	C7—N2—H2A	125.2
C15—C16—C17	117.37 (16)	C5—N2—H2A	125.2
C15—C16—H16	121.3	C11—N3—C17	106.47 (13)
C17—C16—H16	121.3	C11—N4—C12	108.91 (13)
C16—C17—C12	120.65 (15)	C11—N4—H4A	125.5
C16—C17—N3	130.69 (14)	C12—N4—H4A	125.5
C12—C17—N3	108.65 (14)	C33—O1—H1	109.5
C19—C18—C23	119.38 (14)	C35—O4—H4	109.5
C19—C18—C24	120.47 (14)	C34—O6—H6	109.5
C23—C18—C24	120.14 (15)	C24—O7—H7	109.5
C18—C19—C20	119.91 (15)	C25—O9—H9	109.5
C18—C19—H19	120.0	H2—O1W—H3	105.4
			
C6—C1—C2—C3	−0.1 (3)	C21—C20—C25—O9	−177.31 (16)
C1—C2—C3—C4	0.7 (4)	C19—C20—C25—O9	3.7 (3)
C2—C3—C4—C5	−0.3 (3)	C23—C22—C26—O11	−176.01 (16)
C3—C4—C5—N2	−178.11 (19)	C21—C22—C26—O11	4.1 (2)
C3—C4—C5—C6	−0.5 (3)	C23—C22—C26—O12	3.1 (2)
C2—C1—C6—N1	178.2 (2)	C21—C22—C26—O12	−176.72 (17)
C2—C1—C6—C5	−0.7 (3)	C32—C27—C28—C29	−0.6 (3)
C4—C5—C6—C1	1.1 (3)	C33—C27—C28—C29	178.26 (17)
N2—C5—C6—C1	179.23 (16)	C27—C28—C29—C30	0.8 (3)
C4—C5—C6—N1	−178.06 (17)	C27—C28—C29—C34	−178.12 (15)
N2—C5—C6—N1	0.1 (2)	C28—C29—C30—C31	−0.7 (2)
N1—C7—C8—C9	−104.6 (2)	C34—C29—C30—C31	178.24 (15)
N2—C7—C8—C9	72.0 (2)	C29—C30—C31—C32	0.4 (2)
C7—C8—C9—C10	72.0 (2)	C29—C30—C31—C35	−178.30 (15)
C8—C9—C10—C11	−64.1 (2)	C28—C27—C32—C31	0.3 (3)
C9—C10—C11—N3	−48.0 (3)	C33—C27—C32—C31	−178.63 (16)
C9—C10—C11—N4	131.08 (18)	C30—C31—C32—C27	−0.2 (3)
N4—C12—C13—C14	−178.6 (2)	C35—C31—C32—C27	178.58 (15)
C17—C12—C13—C14	−1.0 (3)	C32—C27—C33—O2	4.6 (3)
C12—C13—C14—C15	−0.7 (3)	C28—C27—C33—O2	−174.27 (18)
C13—C14—C15—C16	1.8 (3)	C32—C27—C33—O1	−175.43 (17)
C14—C15—C16—C17	−1.0 (3)	C28—C27—C33—O1	5.7 (3)
C15—C16—C17—C12	−0.6 (3)	C30—C29—C34—O5	1.0 (3)
C15—C16—C17—N3	178.61 (17)	C28—C29—C34—O5	179.92 (18)
N4—C12—C17—C16	179.87 (16)	C30—C29—C34—O6	−178.12 (15)
C13—C12—C17—C16	1.7 (3)	C28—C29—C34—O6	0.8 (2)
N4—C12—C17—N3	0.49 (18)	C32—C31—C35—O3	6.9 (2)
C13—C12—C17—N3	−177.72 (16)	C30—C31—C35—O3	−174.34 (16)
C23—C18—C19—C20	1.2 (2)	C32—C31—C35—O4	−173.69 (16)
C24—C18—C19—C20	−177.37 (15)	C30—C31—C35—O4	5.0 (2)
C18—C19—C20—C21	−2.2 (3)	N2—C7—N1—C6	−1.6 (2)
C18—C19—C20—C25	176.72 (17)	C8—C7—N1—C6	175.51 (17)
C19—C20—C21—C22	1.4 (3)	C1—C6—N1—C7	−178.09 (19)
C25—C20—C21—C22	−177.55 (17)	C5—C6—N1—C7	0.9 (2)
C20—C21—C22—C23	0.3 (3)	N1—C7—N2—C5	1.7 (2)
C20—C21—C22—C26	−179.84 (15)	C8—C7—N2—C5	−175.36 (17)
C21—C22—C23—C18	−1.3 (2)	C4—C5—N2—C7	176.8 (2)
C26—C22—C23—C18	178.84 (15)	C6—C5—N2—C7	−1.1 (2)
C19—C18—C23—C22	0.5 (2)	N4—C11—N3—C17	−1.25 (19)
C24—C18—C23—C22	179.15 (15)	C10—C11—N3—C17	177.92 (16)
C19—C18—C24—O8	−11.2 (3)	C16—C17—N3—C11	−178.86 (18)
C23—C18—C24—O8	170.21 (16)	C12—C17—N3—C11	0.44 (18)
C19—C18—C24—O7	167.73 (15)	N3—C11—N4—C12	1.6 (2)
C23—C18—C24—O7	−10.9 (2)	C10—C11—N4—C12	−177.60 (16)
C21—C20—C25—O10	3.5 (3)	C13—C12—N4—C11	176.7 (2)
C19—C20—C25—O10	−175.51 (18)	C17—C12—N4—C11	−1.24 (19)

### Hydrogen-bond geometry (Å, °)

**Table:**

*D*—H···*A*	*D*—H	H···*A*	*D*···*A*	*D*—H···*A*
O1—H1···O10^i^	0.82	1.82	2.6404 (16)	178
O9—H9···O2^i^	0.82	1.85	2.6682 (16)	177
N1—H1A···O11	0.86	1.84	2.6560 (17)	159
N4—H4A···O3	0.86	2.05	2.8435 (17)	153
C1—H221···O10	0.93	2.51	3.414 (2)	163
C9—H9A···O5^ii^	0.97	2.44	3.346 (2)	156

Symmetry codes: (i) −*x*+1, −*y*+1, −*z*; (ii) *x*+1, *y*+1, *z*.
